# Experimental study on the evaluation in thermal-hydraulic-mechanical-chemical properties of cement-stabilized clay

**DOI:** 10.1371/journal.pone.0313760

**Published:** 2024-11-21

**Authors:** Zihan Chen

**Affiliations:** Faculty of engineering, The university of Hong Kong, Hong Kong, China; Henan Polytechnic University, CHINA

## Abstract

A thorough and comprehensive knowledge of the evolutionary pattern in the thermal-hydraulic-mechanical-chemical (THMC) behavior of cement-stabilized clay is essential for a more reasonable design of improved clay in practical engineering. Sensitive marine clays with low strength, reinforced by various cement contents (0%, 6%, and 12%), were produced as samples to study the evolutionary pattern of the THMC behavior of reinforced clays. Samples with designed curing times (1, 3, 7, 28, and 90 days) will be trimmed to the appropriate size and utilized in a series of mechanical tests. Furthermore, the other portion of the samples, which is pre-installed with sensors, is used to monitor the development in internal temperature, volumetric water content, suction, and electrical conductivity for 90 days. The results showed that the content of cement and curing time have a significant influence on the THMC behavior of reinforced clay. Meanwhile, the results indicate a strong coupling of the THMC behavior of clays reinforced by cement. The results will be of critical assistance in gaining advanced knowledge into the evolutionary pattern in the THMC behavior of reinforced clays as well as making a contribution to optimizing the stability, cost-effectiveness, and durability of the design of reinforced clays.

## Introduction

Marine clays are widely distributed in many coastal and offshore areas of the world, such as North America, Europe, Asia, and Australia [1–4]. Given the main components of marine clays are kaolinite, illite, and montmorillonite, they have the characteristics of high natural water content, high compressibility, low strength, and low permeability [5–7]. These poor engineering properties may cause unacceptable settlements and result in infrastructure projects having low bearing capacity, and thus marine clays are not allowed to be served directly as the subgrade. Hence, it is necessary to implement effective clay treatment measures to achieve the target strength that is capable of supporting the construction and pavement structure loading. In general, a series of typical treatment measures including physical stabilization, chemical stabilization, and biological stabilization were applied to improve the strength of clay. Compared to other methods, chemical stabilization is a popular solution due to it is more economic and effective, as well as better-performed [7]. This method can not only take full advantage of the marine clay to meet the strength to support the construction and pavement loading, but also reduce the amount of dust emission as well as the cost of excavation and transportation to achieve the purpose of protecting the environment and saving costs [6, 8].

In the past few decades, researchers and engineers have successfully applied chemical stabilizations, such as ordinary Portland cement, lime, fly ash, and blast-furnace slag, in practice to improve the engineering properties of the soft soils [7, 9–14]. As the oldest chemical stabilizer, cement was introduced to improve the mechanical parameters of soils during the 1940s [6]. Cement has gradually become one of the most popular chemical stabilizers in the last few decades results from that faster strength development and easy availability [15, 16]. Indeed, many researchers have incorporated cement into soft soil to increase its strength, stiffness, and permanence, thereby improving the overall stability of cement-stabilized soil structures [15–21]. The performances of cement-stabilized soil are strongly dependent on various factors, such as the soil properties, amount and type of cement, W/C ratio, time, and condition of curing [6, 8, 22–24]. These factors mentioned above are crucial in terms of the mechanical performance, stability, durability, and environmental performance of cement-clay structures, which are key design criteria of cement-clay structures [25, 26]. Also, these performances are functions of thermal (e.g. temperature, thermal conductivity), hydraulic (e.g. suction, volumetric water content (VWC), and permeability), mechanical (e.g. uniaxial compressive strength (UCS), California bearing ratio (CBR), deformation, and stress-strain), chemical (e.g. pore fluid chemistry, electrical conductivity (EC)) [27]. Indeed, once prepared and placed, the cement-clay structures are encountered strong coupled thermal (T), hydraulic (H), mechanical (M), and chemical (C) (THMC) processes or factors, which has been studied in other cementitious materials (e.g. cemented paste backfill, cemented coal gangue-fly ash backfill, and gelfill) [27–30]. For example, the temperature of the surrounding engineering structures and geological bodies is obviously affected by geographical location and season. Also, it is well-known that cement hydration generates a lot of heat result in the temperature of the cement-clay increase. The development of this internal and external temperature has a significant influence on the cement hydration rate [27], thereby affecting the strength development and mechanical deformation of the cement-clay. Moreover, the cement hydration consumes a large amount of moisture within the cement-clay cause the decrease of the VWC and the increase of suction, and thus influence the effective stress [5]. Hence, the performance of the cement-clay structure is influenced by the coupled THMC behavior. The coupled THMC behavior knowledge is crucial for engineers to assess and predict the performance of the cement-clay structures as well as design cost-effective, stable and durable cement-clay structures.

Given the fact that are mentioned above, comprehensive research should be performed to provide a better understanding of the coupled THMC behavior of cement-clay structures. To date, however, most researchers focused on studying the separate influence of one factor (thermal, hydraulic, mechanical, or chemical) on the behavior of cement-clay. For instance, some researchers performed a series of situ measurements and experimental analysises to study mechanical properties (e.g. UCS, shear strength, and stress-strain) of the cement-clay [6, 15, 31–47] as a result of the mechanical stability is crucial for the cement-clay structures. Also, some existing researches concentrated on studying the influence of the binder type and dosage, water content, and void ratio on the development of the hydraulic process within the treated clay [43, 48, 49]. In addition, the thermal conductivity is considered as the most significant performance which can indicate the capacity to transfer heat within the cement-clay structures, and thus many study efforts have focused on researching the thermal conductivity (i.e. thermal properties) [50–52].

In view of the fact that the application of coupling processes plays a crucial role in solving practical engineering problems, some research efforts were performed to provide a deeper understanding of the coupled processes in the treated soil. Experimental studies and numerical modeling were performed on to have a deeper understanding on the MC [53, 54], HM [55, 56], THM [51], HMC [43, 57], THCM [58] behavior of treat soil. Although the aforementioned studies have made a fundamental understanding of the THMC behavior of the treated soil, there are no comprehensive investigations that have tackled the THMC behaviour of the cement-clay. Hence, the main objective of this study is to determine the THMC behaviours of sensitive marine clay stabilized with cement in column experiments.

## Materials and experimental program

### Materials

The sensitive marine clays, water, and cement were used in this study.

#### Sensitive marine clays

**Fig 1:**
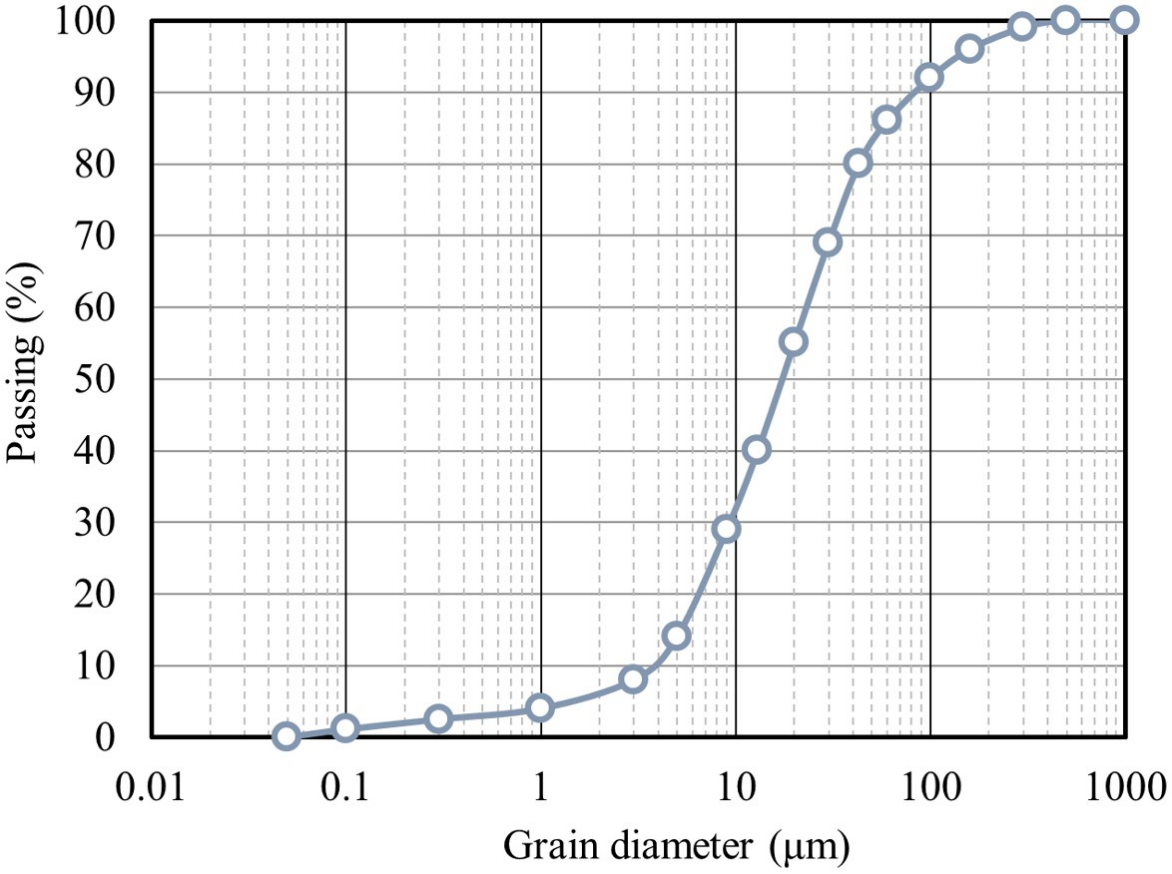
Grain-size distribution of the marine clay.

The sensitive marine clays were collected from a depth of 1.5 to 2.5 meters in the To Kwa Wan typhoon shelter at Kowloon Bay, Hong Kong, where is the representative marine clay distribution locations. The main minerals of this clay include quartz, feldspars, illite, chlorite, and amphibole. The main characteristics of the sensitive marine clays, which were obtained by performing a series of standard laboratory geotechnical tests, were shown in Table 1. Furthermore, the particle size distribution of the sensitive marine clays is illustrated in Fig 1.

**Table 1:** Main properties of sensitive marine clay.

Properties	Specific gravity	Water content	Liquid limit	Plastic limit	Plasticity index	Liquidity index	Optimum water content	dry density at optimum
**Unit**	-	%	%	%	%	-	%	g.cm^-3^
	2.72–2.78	56.6	44.57	15.91	28.42	1.41	18.91	1.43

#### Water and cement

Portland cement type I (PCI), as the most common binder, was applied as the binder in this study. The cement was provided by Green Island Cement; the clinker of cement is mainly produced by the company’s fully integrated manufacturing facility in the Tap Shek Kok plant (Hong Kong). Table 2 lists the typical chemical components of PCI, which are offered by the manufacturer. The mixture ratios of PCI and dry clay used in this study were 0 wt.%, 6 wt.%, and 12 wt.% in order to study the effect of cement content on the behaviour of sensitive marine clay. Distilled water was applied in this study, and the optimum water content was the design moisture content for the column sample preparation. Therefore, the stabilized clay should be performed the standard Proctor compaction test to obtain its maximum dry density and optimum water content before the column sample preparation.

**Table 2:** The chemical components of PCI.

Component unit	SO_3_ (wt.%)	Fe_2_O_3_ (wt.%)	Al_2_O_3_ (wt.%)	SiO_2_ (wt.%)	CaO (wt.%)	MgO (wt.%)
**PCI**	3.82	2.70	4.53	18.03	62.82	2.65

### Preparation program for the sample

A total of eighteen column samples with a constant moisture content (18.91%), cement content of 0 wt.%, 6 wt.%, and 12 wt.% were prepared. The undisturbed clays were immediately put into a sealed barrel after being excavated from the site, which in order to obtain typical geotechnical characteristics of undisturbed clays by carrying out a series of standard laboratory geotechnical tests. Prior to all the tests, the clays were dried in an oven with a constant temperature at 105°C for 24 hours to eliminate their water. The dried clays were then ground into powder with a wooden hammer until 100% pass a 2-mm sieve. Next, the dried clay powders were thoroughly mixed with the design amount of cement (i.e., 0, 6%, 12%) for about 5 min to achieve a uniform mixture. After that, the calculated amount of water was added to the cement-clay mixture were stirred for 5 min to obtain a thorough and homogeneous mixing. After the calculated amount of treated-clay mixtures were produced, they were placed into a mold with a height of 300 mm and a diameter of 152mm in six layers. It should be emphasized that each layer of the mixture should be compacted to set height (i.e. 50cm) and then grooves should be carved on the surface of each layer to improve the connectivity of the contact surface. The prepared specimens were wrapped with plastic film to prevent moisture from evaporating, and they were placed in bigger tubes with a constant temperature and humidity.

### Experimental setup of the column experiments

**Fig 2:**
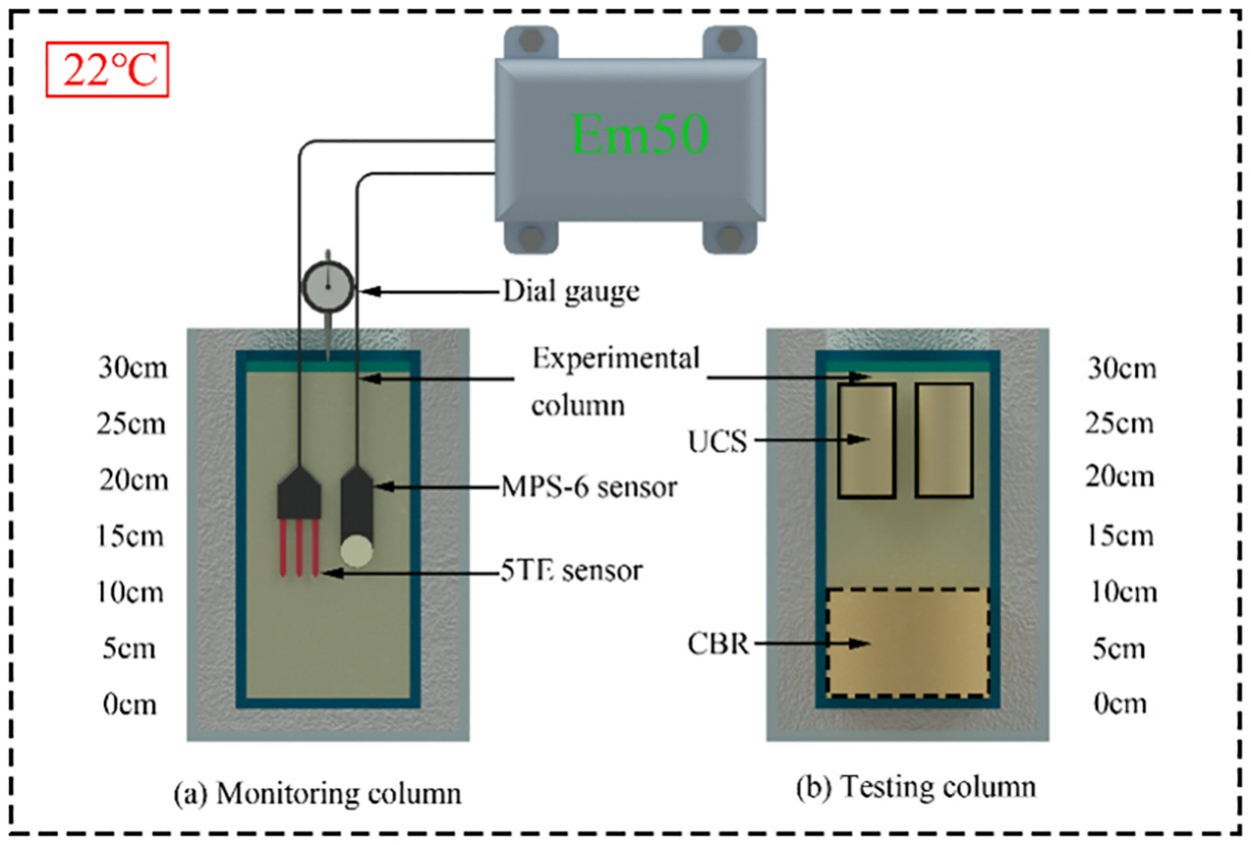
Experimental setups of the monitoring column and testing column experiments.

Fig 2 shows the experimental setup of the column experiments. The columns were made of PVC form tubes with a height of 300 mm and an inner diameter of 152mm. In order to limit thermal interactions between the stabilized clay and the surrounding environment, the column was placed in a bigger column with an inner diameter of 250mm, and the expansive insulating foam has filled the interspace between the two columns. For the purpose of monitoring and sampling, a total of eighteen column samples were manufactured, which include three columns for monitoring (Fig 2a) and the other fifteen columns for preparing the specimens (Fig 2b) to perform various tests. In order to study the influence of cement content on the THCM behaviour of stabilized clay, the three monitoring column samples with different cement contents (i.e. 0 wt.%, 6 wt.%, and 12 wt.%) cured 90 days. The testing columns contained 0 wt.%, 6 wt.%, or 12 wt.% PCI were cured in a curing chamber with constant temperature (22°C) and humidity for 1, 3, 7, 28, and 90 days, respectively. Once the specified curing times arrived, the testing column sample was taken out from the molds. The specimens of specified size for the various engineering tests were taken out from the testing column samples. The required specimens for the various laboratory tests, including uniaxial compressive strength (UCS), California Bearing Ratio (CBR), saturated hydraulic conductivity tests were trimmed into specified size and shape.

### Monitoring program of the column experiments

The Dielectric Water Potential Sensor (MPS-6) and ECH_2_O 5-TE sensor were installed at centre of the monitoring columns (Fig 2a) to continuously monitor the evolution of the suction, temperature, electrical conductivity (EC), and volumetric water content (VWC) after column filling. The monitoring of suction and VWC can contribute to reveal the influences of cement content and curing time on the evolution in the self-desiccation of stabilized clay. The changes in the monitoring temperature of treated clay result from the heat released by binder hydration, thus it can provide a better understanding of the influence of cement content on the rate of binder hydration. The monitoring of EC is an effective method to evaluate the progress of cement hydration and track the changes in the internal microstructure of the hydrating cementitious materials [59]. The evolution of matric suction and temperature with time were recorded by using an MPS-6 sensor, while a 5TE sensor was applied to record the EC, VWC, and temperature. The sensors were connected to an EM50 data logger to collect data. In addition, a dial gauge was installed at the top immediately after the last layer clay was compacted to monitor vertical settlement results from self-weight and binder hydration.

### Experimental test program

#### Mechanical tests (UCS, CBR test)

**Fig 3:**
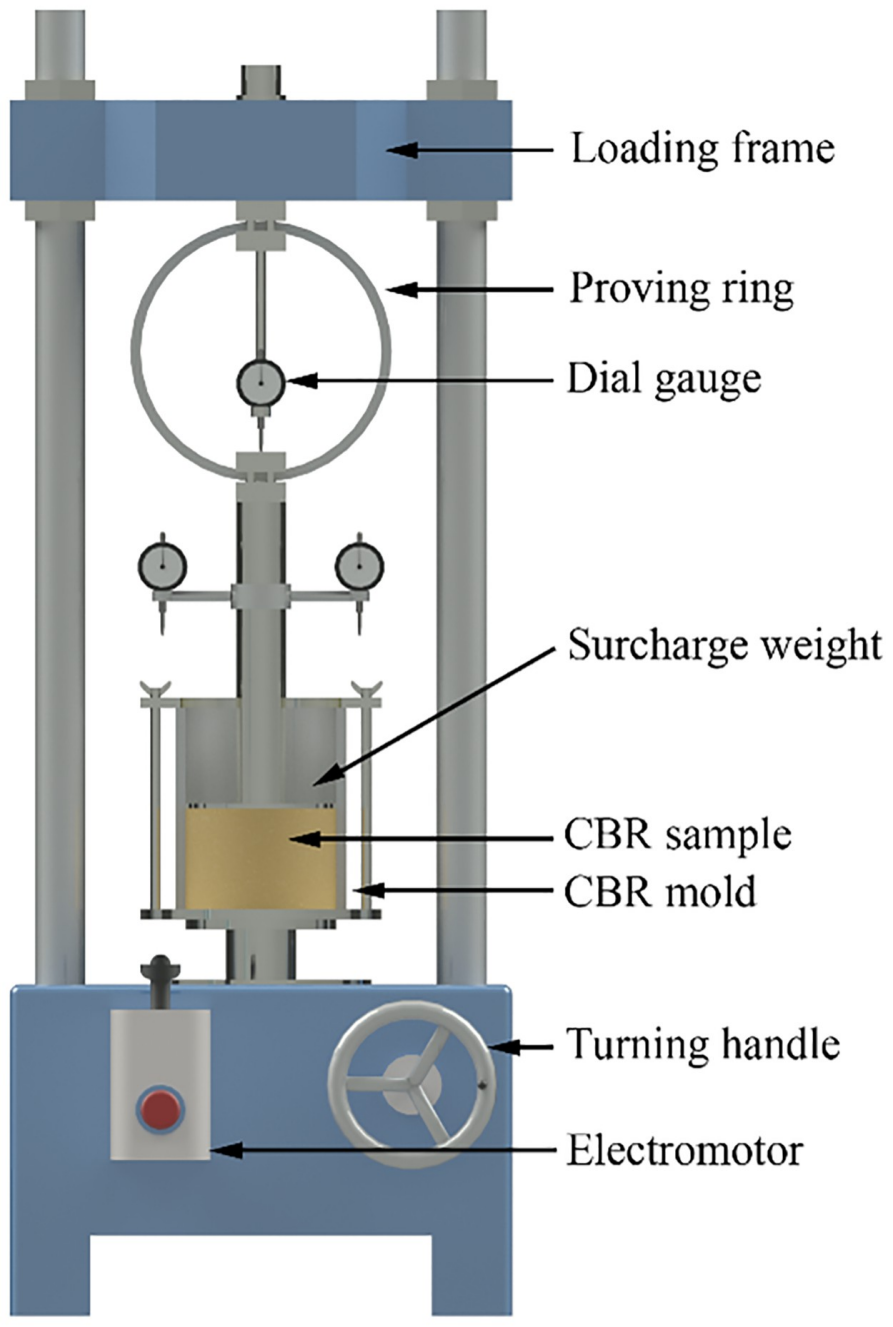
Setup of the CBR test.

**Fig 4:**
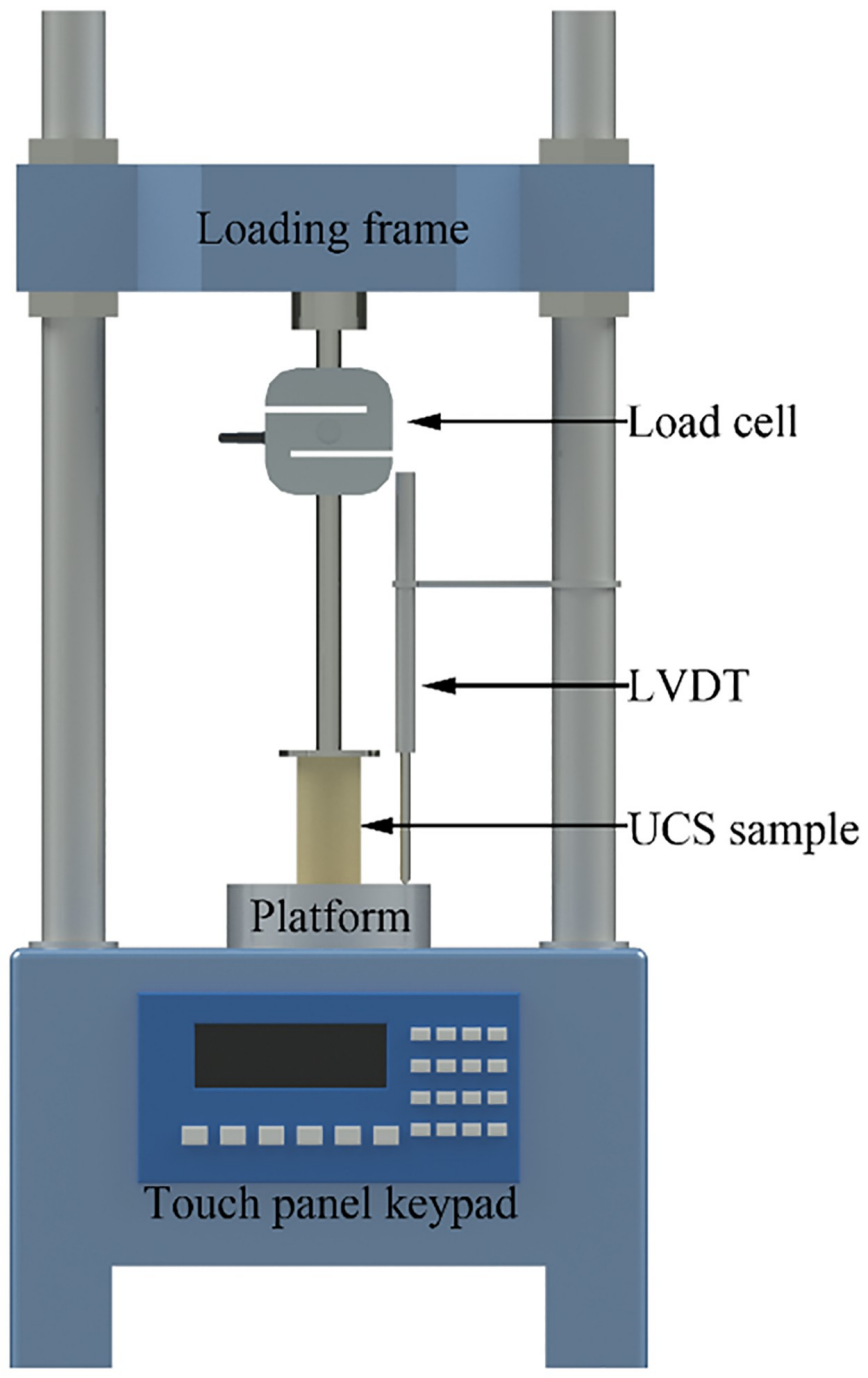
Setup of the UCS test.

The CBR test (ASTM D1883) and UCS test (ASTM D5102) were performed on all of the samples taken out from the testing column after a specified curing time. The sample for the test would be physically cut out of the testing column and polished to a smooth and suitable size. The schematic diagram of the CBR test setup and UCS test setup are illustrated in Figs 3 and 4, respectively.

The UCS test is extensively used to determine the mechanical stability of intact/remolded clay structures due to it is relatively inexpensive and quick. The UCS test was carried out by the Digital Tritest 50, which has a loading capacity of 50kN. The UCS test was performed at a constant loading rate of 1mm/min according to ASTM D5102. It should be noted that the test was repeated a minimum of two times to ensure the repeatability of the experimental results.

The CBR test is a well-known way to evaluate the bearing capacity of subgrades [60]. The sample with a height of 118.5mm as well as a diameter of 152mm was taken out from the bottom of the testing column was performed the one point CBR test. Generally, the CBR is the specific value of the unit load on the piston at 2.5 mm penetration to the unit load calculated to penetrate a standard material of well-graded crusted stone. The test was performed at a constant penetrate rate of 1.25 mm/min according to ASTM D1883.

#### Saturated hydraulic conductivity tests

The TRI-FLEX II was utilized to measure the saturated hydraulic conductivity of the stabilized clay specimens. The related procedure for this test is described in detail in ASTM D5084, and the test was carried out in the constant head model. The specimens were soaked in water for 12 hours before the test. The test continued until the saturated hydraulic conductivity was steady. Each test was repeated at least two times and the mean value was considered as saturated hydraulic conductivity.

#### Thermal conductivity tests

The thermal conductivity tests were performed by applying the KD2 Thermal Properties Analyser, which obtains the thermal conductivity by recording the dissipation in heat from a line source given a known voltage. The thermal conductivity test was carried out in according with the operator’s manual for KD2 Pro Thermal Properties Analyzer. The dual needle SH-1 sensor was used in this research due to it is a good choice for stabilized clay. Prior to the test, two holes with a diameter of 2.5mm were drilled for the dual needle SH-1 sensor, and a silver polysynthetic compound was filled the gap between the needle probe and walls of the hole in order to ensure close-connected between them.

## Discussions and results

### Evolution of the hydraulic properties (permeability, suction, VWC)

#### Permeability

**Fig 5:**
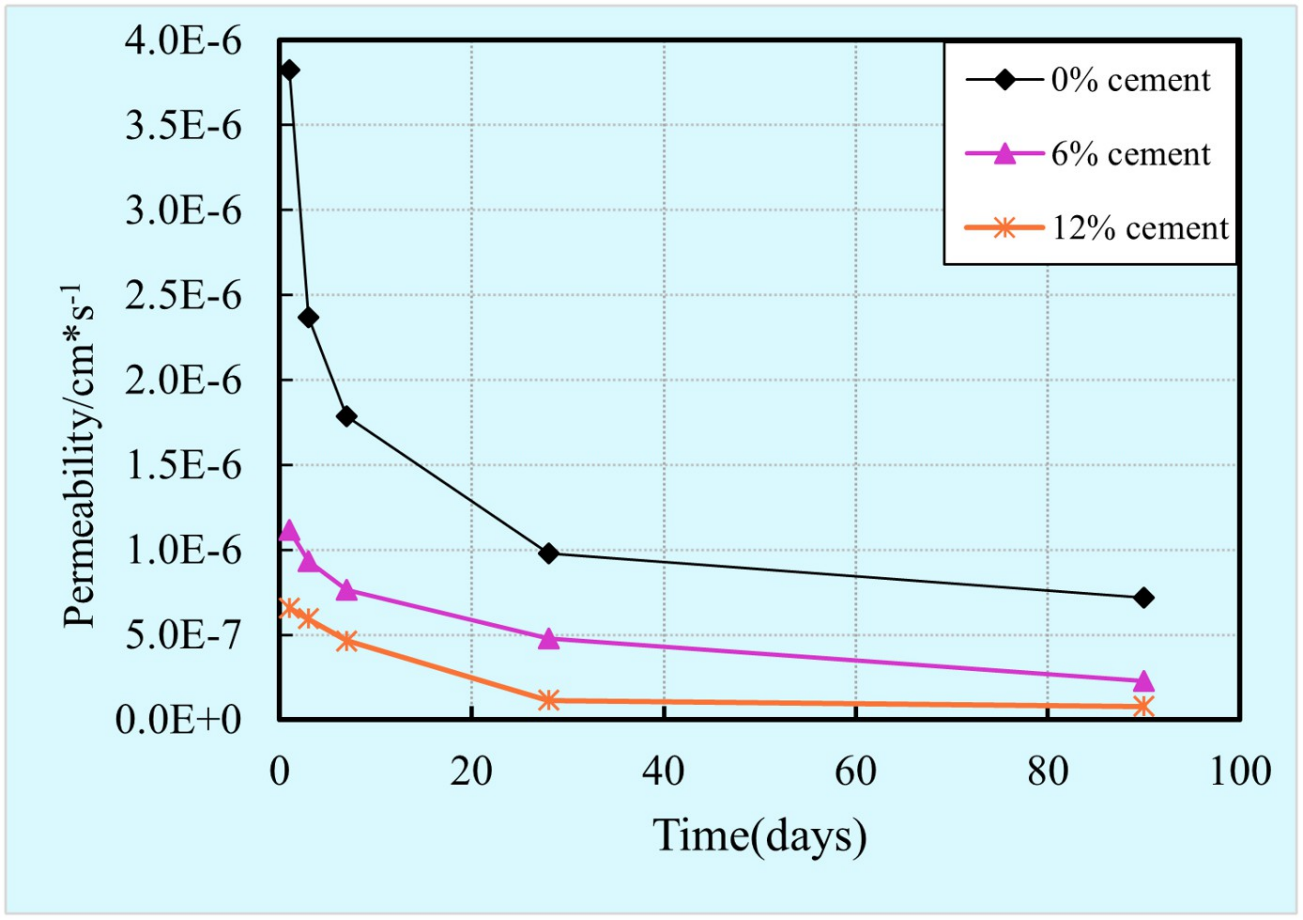
Development of permeability with cement content and curing time.

The evolution of permeability with the cement content and curing time are illustrated in Fig 5. It can be obviously observed from this figure that for any amount of cement content, the permeability decreases as the curing time increases. All curves show a non-linear reduction, it is clearly that the reduced rate of permeability is obviously higher at the initial stage than that of the later period. This behavior is mainly attributed to the fact that the degree of cement hydration increase as time goes by, as a result, more hydration products are generated in the mixtures [61]. These cement hydration products precipitate in the pores resulting in a further refinement of the pore structure and a decrease in porosity. Thus, the permeability decreases as time increases. This finding is in accordance with that occurs in other cementitious materials [32].

From Fig 5, it can be found that the cement content has a significant effect on the permeability. The permeability reduces as a larger amount of cement add into the stabilized clay. This is due to the fact that a higher cement content leads to more hydration products, which precipitate in the stabilized clay matrix result in the refinement of the pore structure. This finding is consistent with the results of previous research on the pore structure of cementitious materials that directly influence their permeability [62]. Indeed, the refinement of pore structure induces the porosity decrease, and thus resulting in permeability reduction. In terms of the evolution of the decrease rate of permeability with the cement content, it is clear that the decrease rate reduces with the cement content. For example, the permeability for the 1 day sample is 3.83 E-6, 1.12E-6, and 6.57E-7 cm/s at the cement content of 0, 6%, and 12%, respectively.

#### Suction

**Fig 6:**
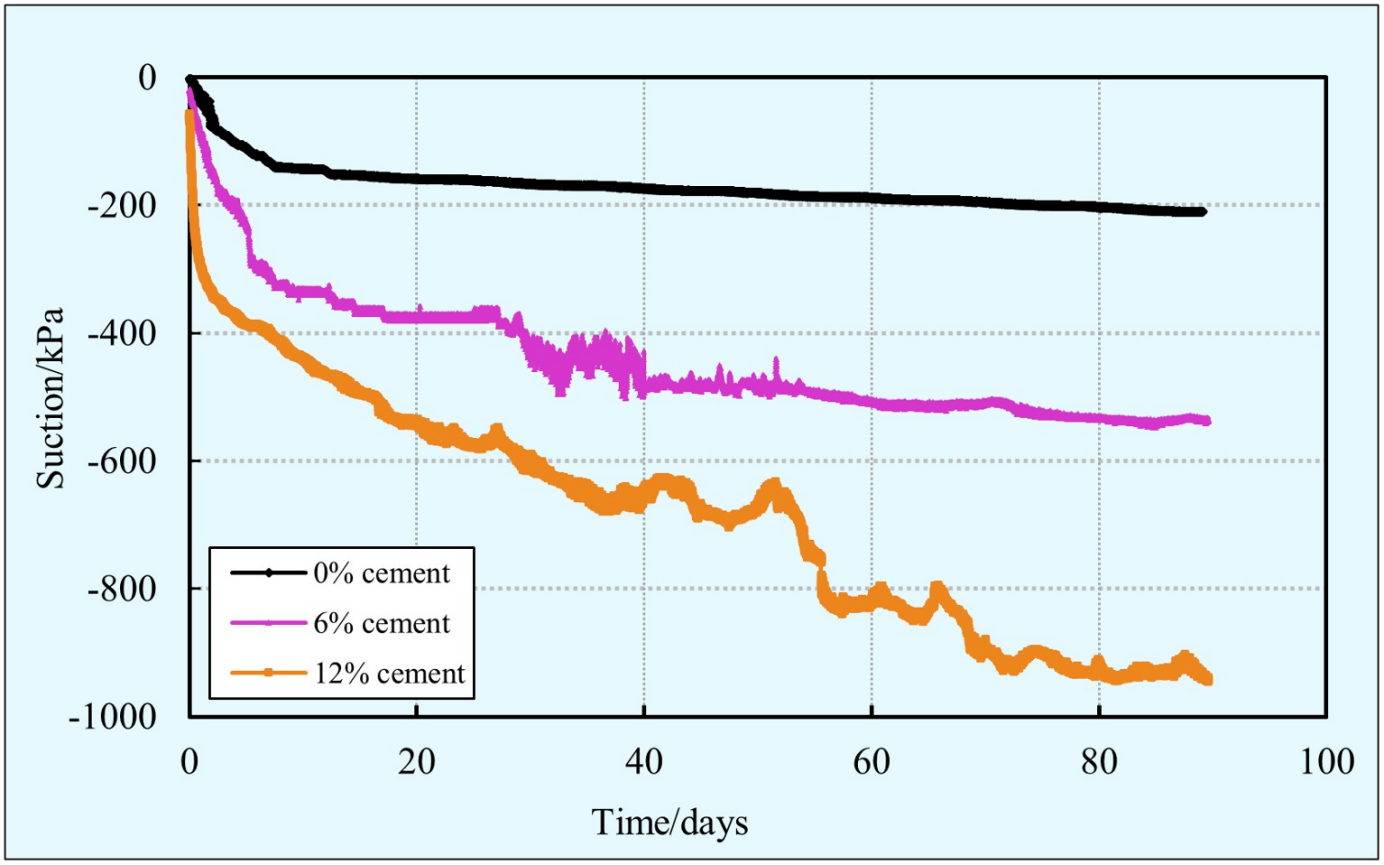
Evolution of suction with cement content and curing time.

The suction evolution of stabilized clay with various cement contents (i.e. 0, 6%, and 12%) is graphically presented in Fig 6. From this figure, it is obvious that the cement content and curing time play a significant role in the suction. All the curves have a similar trend, the suction increases rapidly at an early stage while slows down at later stage. This is due to the fact that the cement and water react quickly to form hydration products once the cement is in contact with water. As time goes by, the formation of a film on the surface of cement particles hinders further cement hydration, as a result, the rate of cement hydration decreases and the consumption of water in the pores is slower. In addition to the aforementioned above, it is also clear that a higher cement content corresponds to a higher suction at a given curing time. As the cement content increases, more hydration products are generated, which leads to more clay particles agglomerate together, and changes the sample particles size, void size, shape, and distribution [63]. As a result, the voids volume and porosity are lower, and thus it is more difficult for gas to enter the samples. Consequently, the suction increases with the increase of the cement content.

#### Volumetric water content (VWC)

**Fig 7:**
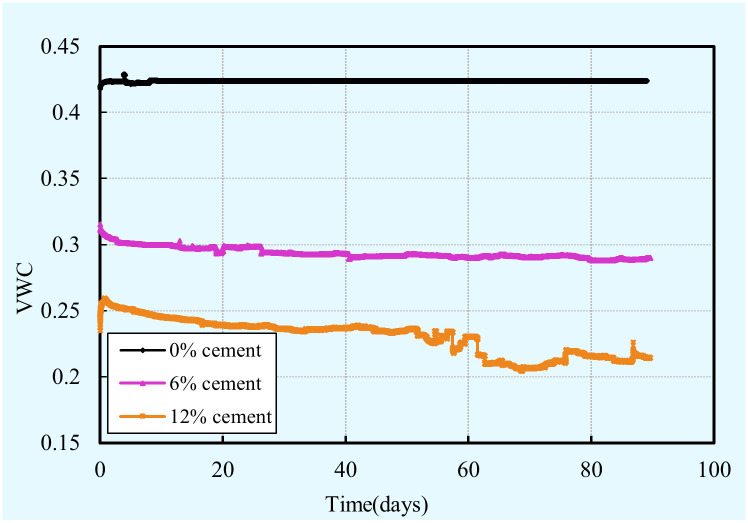
Evolution of VWC with cement content and curing time.

Fig 7 shows the evolution of the volumetric water content (VWC) versus curing time with respect to the cement content. From this figure, it is clear that a significant decrease in VWC with the cement content increase for a given curing time. In other words, a sample with a higher cement content consumes more water. This can be explained by the fact that a high cement content increases the probability of contact between cement and water, thereby leading to the cement hydration is more rapid and intense, as a result, more moisture was consumed. Moreover, it is clear that, regardless of the cement content, the VWC increases up to a maximal value and then decreases with time. This can be mainly attributed to that the sensor is in closer contact with the sample particles with time. The VWC decrease with time can be explained by the fact that more cement hydrates as time increases results in more water were consumed. Furthermore, it is also interesting to note that the VWC of the samples with none cement decreases slightly with time. This implies that evaporation has a slight influence on the VWC. In other words, the evolution of VWC dependent on the combined influence of binder hydration and evaporation, while binder hydration plays a dominant role.

### Evolution of the mechanical properties (UCS, CBR, stress-strain behaviour)

#### Unconfined compressive strength (UCS)

**Fig 8:**
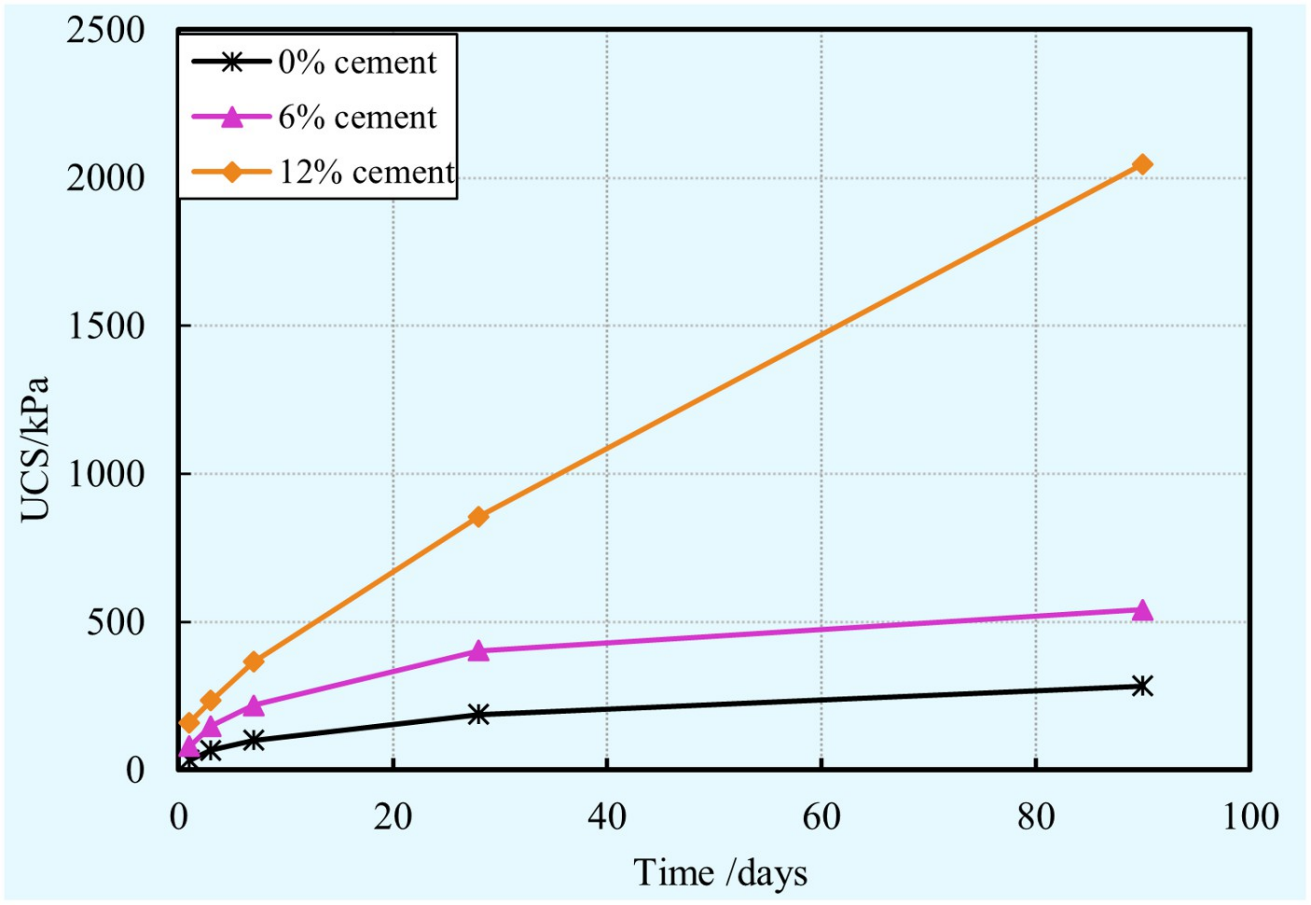
Combined influence of curing time and cement content on the UCS of the cement-clay.

The samples with different cement contents (i.e. 0, 6%, and 12%) were subjected to the unconfined compression test after various curing times (1, 3, 7, 28, and 90 days). The variations in UCS of specimens with different cement contents and cured at various curing times are presented in Fig 8. It is clear that the cement content and curing time play a significant role in terms of the strength development of the stabilized clay samples. These findings reveal that the increase of the curing time is responsible for the increase of UCS. In other words, regardless of the cement content, the UCS of stabilized clay increases with the curing time increases. These time-dependent changes in UCS are as a result of the combined effect of the increased cement hydration and the development of suction. Indeed, a longer time responds to a higher amount of hydration products, such as calcium-silicate-hydrate (C-S-H), calcium hydroxide (CH). Furthermore, C-S-H is deemed to be a primary binding phase in hardened cement [64], which is a main contributor to the strength increase. From Fig 2, it is interesting to notice that the rate of stabilized clay UCS gain before 7 days of curing is higher than that at advanced ages. This observation is closely associated with the properties of cement. It is because of that the hydration rate of cement is faster in the early stage, and thus resulting in hydration products were formed faster.

From Fig 8, it is clear that the UCS increases with the cement content increases for a given curing time. This observation is consistent with the results obtained by [65, 66]. It is well-known that a sample with higher cement content generates more hydration products. As mentioned previously, a higher amount of hydration products is beneficial for the UCS increases. This is primarily result from the combined effects of two factors: (a) a higher cement content caused a more rapid and intense hydration reaction rate; (b) a higher cement content caused more intense self-desiccation. A sample with a higher cement content generates more hydration products, which was evidenced by carrying out the mercury intrusion porosimetry (MIP) test and scanning electron microscopy (SEM) test [31]. More hydration products contribute to increase the strength of the cement-clays due to calcium silicate hydrate (one of the main hydration products) is deemed to be a primary binding phase in hardened cement [64]. The hydration products bind the clay particles together and generate a stronger clay matrix [33, 42, 43]. Consequently, more hydration products lead to a stronger unconfined compressive strength as the cement content is increased. Furthermore, this higher amount of hydration products caused the refinement of the pore structure due to these hydration products (e.g., ettringite, gypsum) precipitate in the empty capillary pores of the cement-clays. Moreover, the refinement of the pore structure contributes to strength gain [67]. Usama Khalid et al. [31] performed SEM and MIP tests demonstrated that the total pores volume decrease with cement content increase. In addition, more intense self-desiccation contributes to more cement result in strength increase of cement-clay. This is mainly attributable to that suction development can caused strength increase of unsaturated porous media [68].

#### California bearing ratio (CBR)

**Fig 9:**
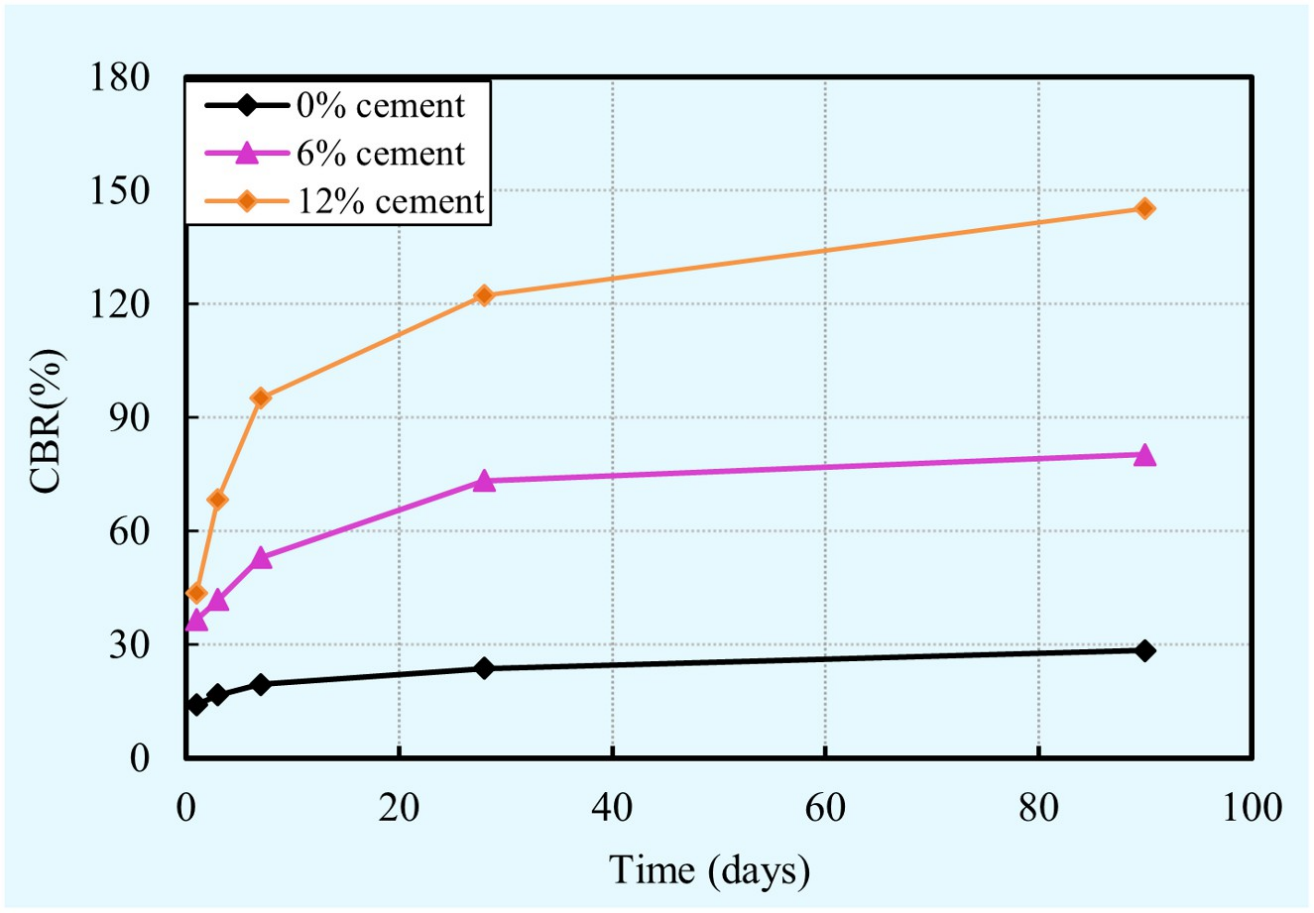
Combined influence of curing time and cement content on the CBR of the cement-clay.

CBR is considered as a most useful parameter to design pavement in the last few decades [69, 70], and thus 30 specimens were performed on the CBR test with an aim to reveal the effect of cement content on the CBR value of cement-clay. The evolution of the CBR value of the cement-clay samples with various cement contents is illustrated in Fig 9. From this figure, it is obvious that the CBR value of cement-clay depends on combined effects the cement content and curing time.

It can be found that in spite of the curing time, the CBR value increase as the cement content increase. This is mainly attributed to that higher amounts of cement result in more hydration products were formed and more moisture were consumed. The reason for this is analyzed in the previous part. More hydration products were formed in the sample to improve the structure of thes ample. In other words, a higher cement content causes the void ratio to decrease and strengthens the interaction between clay particles. As a result, the bearing capacity increases as the cement content increases. In other words, the CBR value increases with increasing cement content. On the other hand, more cement consumes more water in the capillary pores results in the water content decreases and the suction increases. However, it should be mentioned that the water content and the suction play an important role in determining the CBR value of cement-clay. According to the results of previous studies [71–73], the CBR value increases with decreasing moisture content and increasing suction. Furthermore, it is also obviously found that, regardless of the cement content, the CBR value with the increase of curing time. This finding is mainly attributed to the fact that more hydration products were generated and more moisture was consumed as the curing time goes up. As explained previously, this is because more hydration products cause the void ratio to decrease and strengthen the interaction between clay particles, in addition, the CBR value increases with decreasing moisture content and increasing suction.

#### Stress-strain behaviour

**Fig 10:**
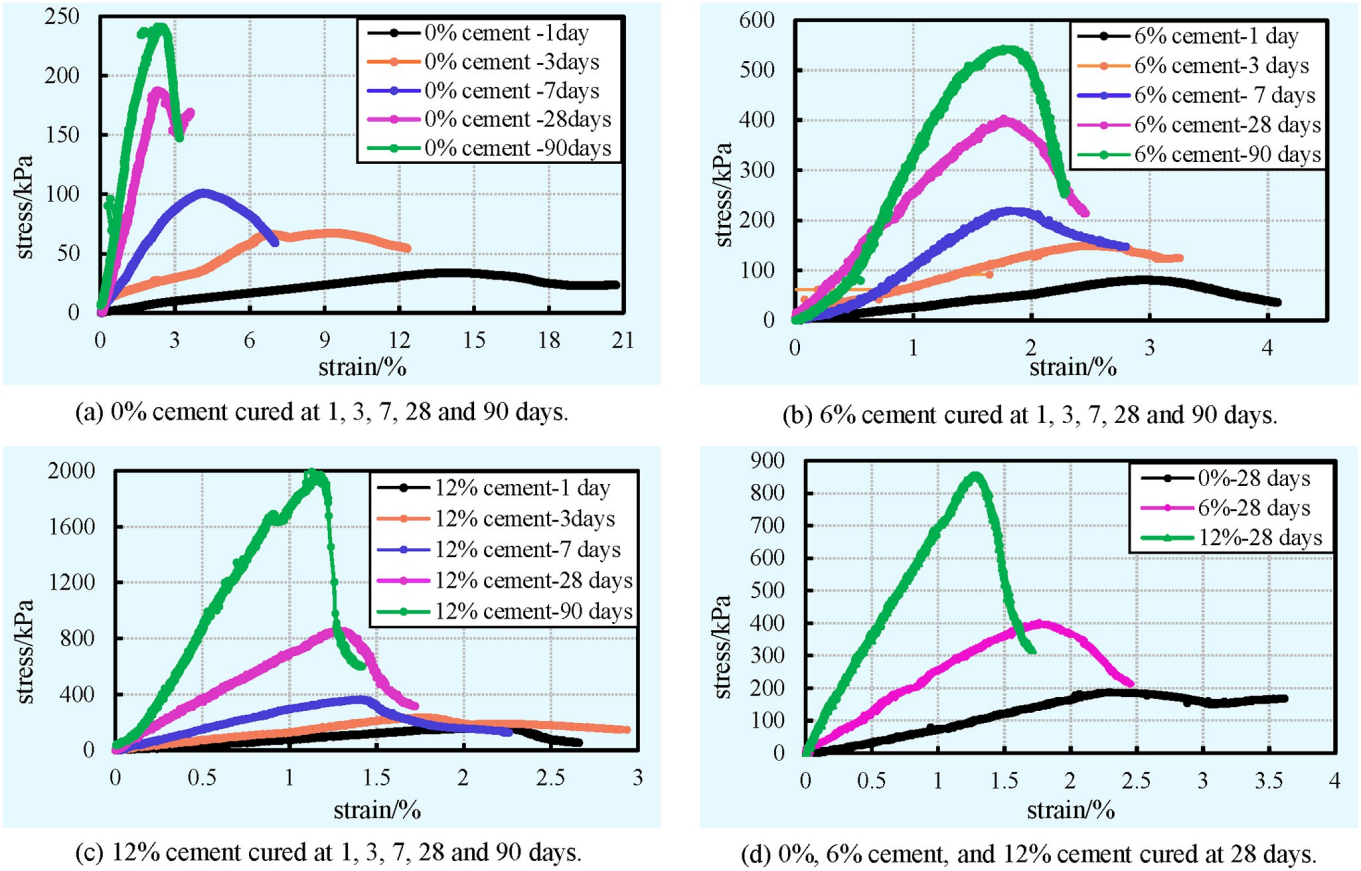
Influence of curing time and cement content on stress-strain behaviour of cement-clay. (a) 0% cement. (b) 6% cement. (c) 12% cement cured at 1, 3, 7, 28 and 90 days. (d) 0%, 6% cement, and 12% cement cured at 28 days.

In order to investigate the stress-strain behaviour of cement-clay samples with different cement contents (0%, 6%, and 12%) at various curing times (1, 3, 7, 28, and 90 days), approximately 45 cement-clay specimens were subjected to the uniaxial compression test. Multiple sets of typical stress-strain curves obtained from the UCS tests on cement-clay samples with different cement contents at various curing days are presented in Fig 10, the UCS and the average modulus of elasticity (E_50_) are shown in Table 3. It can be obviously found that, in spite of the curing time and cement content, all of the stress-strain curves show similar hump curve shapes, with an increase up to the peak stress and then reduce. These curves can be used to determine the failure strain, deformation modulus E_50_, and peak stress (i.e. UCS) [74]. However, these parameters and the shape of the stress-strain curve are significantly affected by the cement content and curing time. It can be clearly seen that the peak stress and deformation modulus of the cement-clay increase with the cement content, while the failure strain decreases (Fig 10d). It can be clearly observed in Fig 10d that the growth rate of stress before the peak stress (upward slope) and the reduction rate of stress with strain after the peak stress (downward slope) increase with the increase of cement content. This means that a sample with more cement has higher stiffness and shows more brittle deformation. In other words, a larger amount of cement causes a change in the mode of failure of cement-clay samples. All the phenomena mentioned above can be mainly attributed to the fact that higher cement content results in more cement hydration products were generated in the cement-clay matrix. Indeed, it is well known that more hydration products result in a denser and more stable structure and a higher strength of cement-clay, which has been analyzed in the previous section. Moreover, a denser and more stable structure causes a more brittle deformation, higher stiffness, and deformation modulus, while a higher strength corresponds to larger peak stress (i.e. UCS). Furthermore, a higher strength contributes to that the sample can accumulate more energy until the stress with an increase up to the peak value. This phenomenon leads to the cracks propagation and rapid deformation when the specimen is damaged, and thus the stress-strain curve drops rapidly.

**Table 3:** Influence of curing time and cement content on UCS and E50 of cement-clay.

Cement content	Curing time									
	1 day		3 days		7 days		28 days		90 days	
	UCS/Kpa	E_50_/Kpa	UCS/Kpa	E_50_/Kpa	UCS/Kpa	E_50_/Kpa	UCS/Kpa	E_50_/Kpa	UCS/Kpa	E_50_/Kpa
**0%**	34.0	277.1	67.4	877.8	101.1	3170.6	187.4	6935.0	283.5	12673.0
**6%**	80.8	2298.8	148.9	6691.8	218.9	12738.9	403.1	19169.5	542.0	30994.3
**12%**	159.7	7336.2	235.0	13331.2	365.1	24065.2	856.1	52989.4	2046.4	99211.7

### Evolution of the thermal properties (temperature, thermal conductivity)

#### Internal temperature

**Fig 11:**
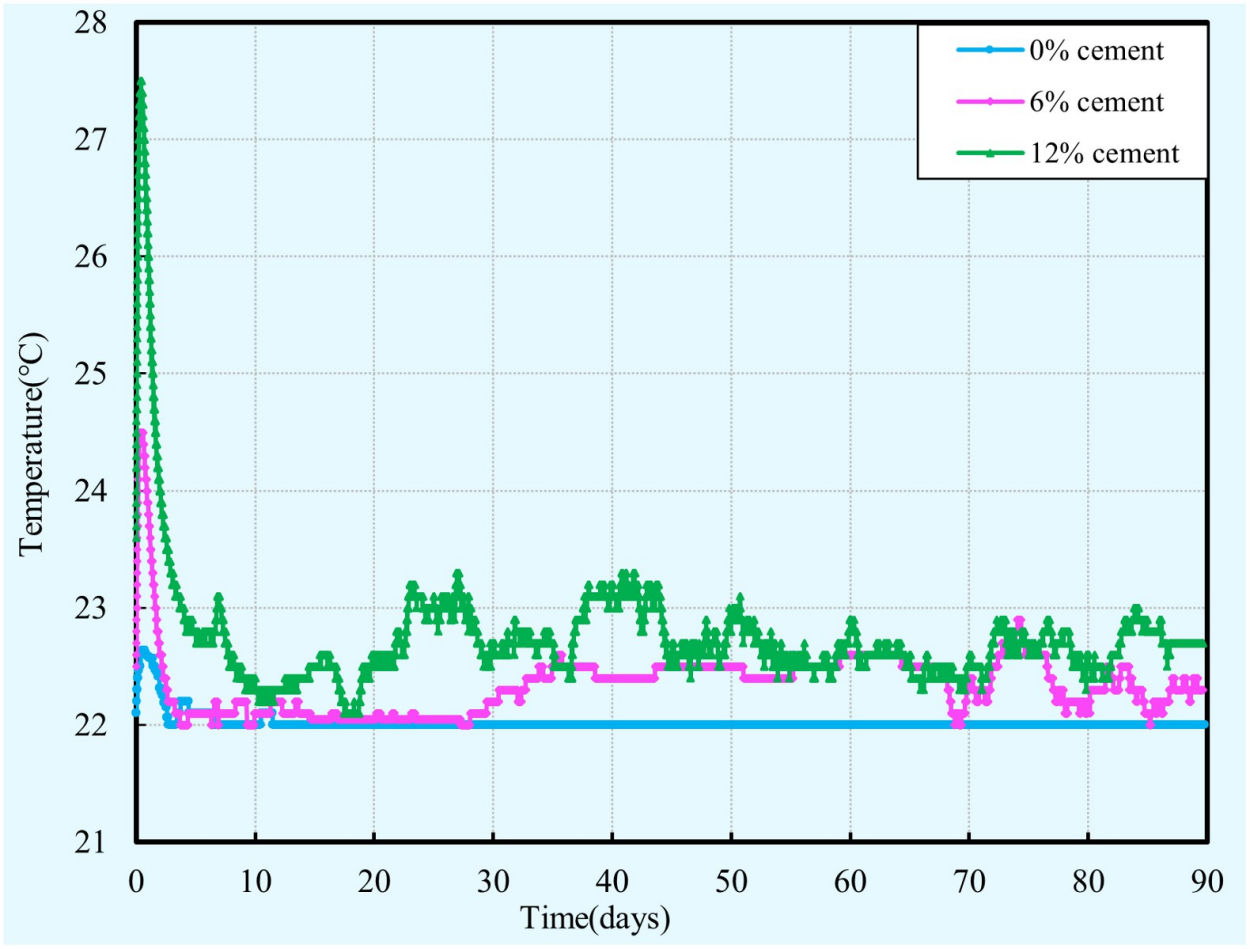
Evolution of temperature within cement-clay with various cement contents.

Fig 11 illustrates the development of the internal temperatures of the cement-clay samples with different cement content during 90 days of curing. These curves have a similar trend, the internal temperature increases at a high rate with time until a peak value occurs, and then decreases until it is consistent with the temperature of the surrounding medium. From Fig 11, it can be seen that the maximum internal temperature of the cement-clay sample increases as the cement content increases. This observation is primarily attributed to the well-known fact that the evolution of the internal temperature of the cement-clay sample is mainly dependent on the heat generated by the cement hydration [27]. Therefore, the growth rate of the internal temperature can be used as an indirect indicator to determine the speed of the hydration reaction. Moreover, more cement react with water will release a larger quantity of heat, this heat cannot dissipate quickly lead to higher internal temperature, and hence the cement-clay sample with more cement has a higher peak internal temperature. Also, it can be seen that the internal temperature of the cement-clay sample with none cement increase with time. This can be explained by the fact that a small amount of heat is generated by the outside work on the clay particles during the sample preparation process.

In addition, it can be concluded that the evolution of the internal temperature of cement-clay structure will influence the strength, suction, hydraulic conductivity, and thermal conductivity of cement-clay, this result is confirmed in other cementitious materials [27]. This can be attributed to the fact that the curing temperature plays an important role in the microstructure and strength of the cementitious material [27]. Moreover, the magnitude of influence is affected by the size of the cement-clay structure, this is because the size of the cement-clay structure has a significant effect on the evolution of temperature within the cement-clay structure. This can be explained by the fact that a larger size cement-clay structure corresponds to more cement is hydrated, as a result, more heat is produced. This heat, in turn, leads to a faster hydration rate. Consequently, higher amounts of hydration products are formed in the cement-clay structure, and hence its hydraulic and mechanical properties are affected.

#### Thermal conductivity

**Fig 12:**
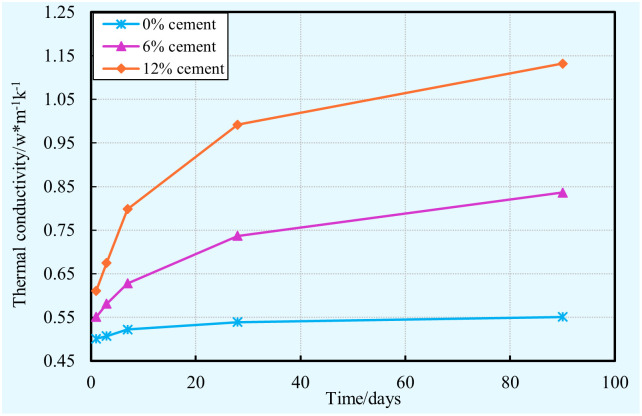
Thermal conductivity evolution of cement-clay with various cement contents.

The variations in thermal conductivity of cement-clay samples with various cement contents for different curing times (1, 3, 7,28, and 90 days) are shown in Fig 12. From this figure, it is obvious that the thermal conductivity of cement-clay samples depends on the combined influence of curing time and cement content. It can be seen that, regardless of the cement content, the thermal conductivity increases as time is increased. This can be explained by the well-known fact that a longer curing time contributes to producing higher amounts of hydration products. These hydration products fill in the pore voids lead to the refinement of pore structure and reduction of the void ratio. Moreover, the self-weight pressure causes a dense matrix, thereby resulting in thermal conductivity of cement-clay sample with none cement increase slightly, as shown in Fig 12. Furthermore, as expected, it is clearly in Fig 12 that in spite of the curing time, the thermal conductivity increases with the cement content increases. It is well-known the fact that a higher amounts of cement result in more hydration products are formed within the cement-clay sample. As a result of the addition of hydration products, the pore structure refines and the void ratio reduces. It is well understood that the refinement of the pore structure and the decrease of the void ratio can result in an increase in the thermal conductivity of cement-clay samples.

### Evolution of the Chemical properties (EC)

**Fig 13:**
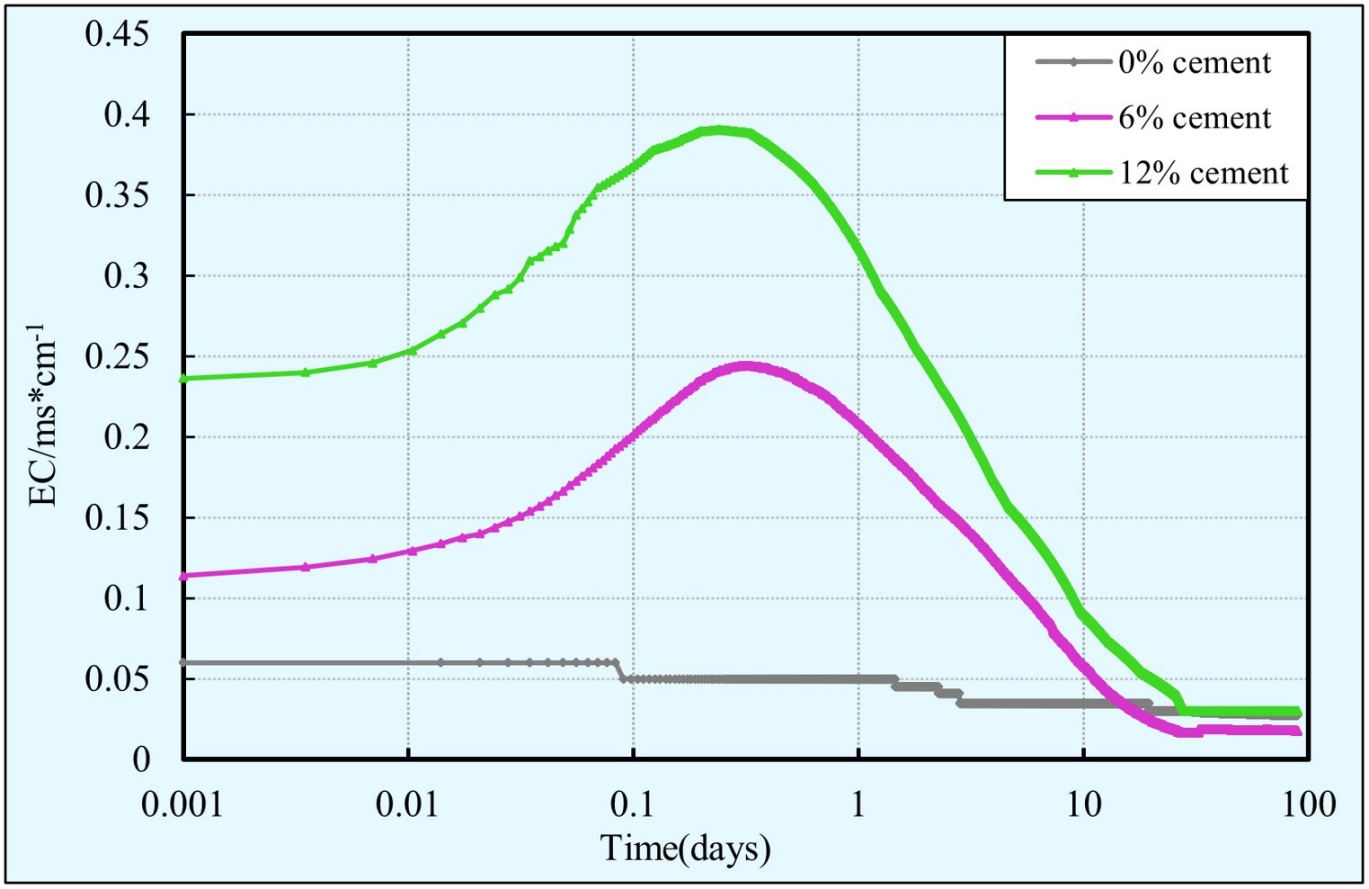
EC evolution of cement-clay with various cement contents.

The cement-clay has the characteristics of electrical conductivity as a result of its interconnected pore network is filled with water containing ions released by cement hydration. The electrical conductivity (EC) of the cement-clay mainly depends on the ion concentration and migration rate in the pore solution. Once marine clays are mixed with cement, a series of reactions occur. However, these reactions are always accompanied by the dissolution of cement components and the formation of hydration products, and thus leading to changes in ion concentration and ion migration rate. Therefore, studying the evolution process of electrical conductivity contribute to a better understanding of the changes in its internal hydration degree, pore structure, and hydration products. Understanding the evolution of the pore structure and hydration products is essential for all the performance characteristics (including strength, volume stability, and durability, etc.) of the cement-clay. Consequently, the electrical conductivity (EC) monitoring was performed on the cement-clay samples with different cement contents for curing 90 days with an aim to have a better understanding of the performance characteristics of the cement-clay, the results of EC monitoring are shown in Fig 13.

It can be seen from Fig 13 that regardless of the amount of cement content, the EC of the cement-clay changes with the curing time in a similar trend, with an increase up to a peak value, then decrease. This phenomenon is mainly attributable to the fact that the dissolution of the cement particle components increases the conductivity of the pore solution, and thus resulting in the EC increases at early age. The initial EC increases slowly mainly because the Ca^2+^, OH^-^ and other trace ions (e.g.: SO_4_^2-^, Na^+^, and K^+^) derive from cement hydration are easily absorbed by the formed thin layer of hydration products. The main components of the thin layer are ettringite and hydrated calcium silicate, and the thin layer wraps around the unhydrated cement particles to prevent the unhydrated cement particles from further reacting to release ions. As the hydration reaction continues, the thin layer is broken, the Na^+^ and K^+^, etc. ions in the cement composition are quickly dissolved, and a large amount of Ca^2+^ and OH^-^ ions are formed. In addition, the ettringite decomposes and releases Ca^2+^ and SO_4_^2-^, resulting in an increase in the number of ions. As a result, the concentration of conductive ions in the pore solution continues to increase, and thus the EC rises rapidly. As the hydration reaction continues, Ca^2+^ ions reach saturation, and dicalcium silicate, tricalcium silicate, etc. rapidly hydrate to form a large amount of hydration products. These hydration products precipitate in the voids lead to a decrease in porosity, which results in changes in the internal structure, a decrease in the availability of ions and free water, and a decrease in the conductive pore solution. Therefore, the formation and accumulation of cement hydrates lead to a large of ions are consumed, resulting in a decrease in ion concentration and a sharp drop in electrical conductivity.

From Fig 13, it can be also clearly observed that the peak EC gradually increases with the increase of the cement content. This is mainly attributed to that the increase in cement content leads to an increase in the probability of contact between cement and water, which increases the degree of hydrolysis of cement components and dissolves more ions, as a result, the peak conductivity increases with the increase of cement content.

### Discussion on the coupled evolution of the THMC processes

**Fig 14:**
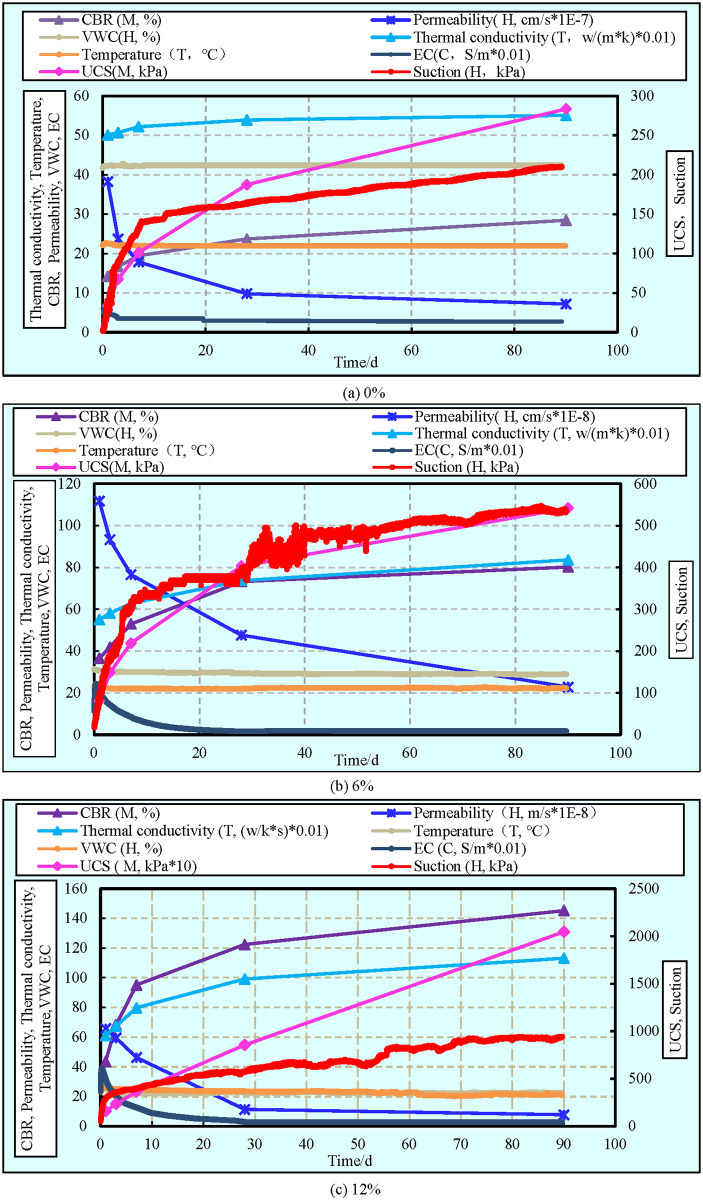
Coupled evolution of the Thermal-Hydraulic-Mechanical-Chemical (THMC) behaviour of cement-clay with various cement contents. (a) 0%. (b) 6%. (c) 12% cement.

A series of monitoring and testing experiments were performed on the cement-clay samples with different cement contents to obtain their thermal (temperature, thermal conductivity; T), hydraulic (permeability, suction, VWC; H), mechanical (UCS, CBR; M), and chemical (EC; C) test results. The simultaneous evolution of these experimental results and the THMC coupled processes are summarized in Fig 14. A better understanding of the THMC coupled processes in clay subgrade stabilized with cement can have important and practical engineering applications in terms of accelerating the engineering project, and the cost-effective design of the clay subgrade.

From Fig 14, it can be seen that the cement content has a significant effect on the evolution of the EC values, which has been detailedly explained in the previous section. Subsequently, more intense hydration (EC as an indication of hydration) corresponding to higher peak internal temperature. This indicates there is a strong coupling between EC and the internal temperature of the cement-clay (C-T coupling behavior). This can be explained by the well-known fact that cement hydration releases a large amount of heat, which leads to an increase in the peak internal temperature. Moreover, it should be noted that the cement content has a significant influence on the C-T coupling behavior. A larger amount of cement corresponding to a higher peak internal temperature and a higher peak EC as explained in the previous part.

In addition, it can be also observed that the C-T coupling plays a significant role in the development of self-desiccation (i.e. suction and VWC) and the strength of the cement-clay. Moreover, the magnitude of the aforementioned influence depends on the cement content of the samples. It is evident in Fig 14 that the samples with higher cement content show higher strength and more intense self-desiccation than those with less cement. Simultaneously, it should be emphasized that Fig 14 shows that there is a strong coupling between the suction and mechanical strength of the sample. It can be also clearly observed that, in spite of cement content increase of suction contributes to the increase of the strength. This finding is in agreement with that higher suction usually leads to higher strength in porous material [68]. From this result, it can be concluded that the higher strength of the sample with a larger amount of cement is not only as a result of more hydration products generated and refinement of the pore structure (as explained in the previous section), but also the increase of suction.

The same trend of increase in UCS and suction with time can be observed through Fig 14, which is particularly evident at the 7-day curing stage. This observation indicates that suction can be effectively employed to assess the strength of the reinforced clay during the initial stages of curing. The rate of increase in suction slows with the passage of time. This is due to the fact that the hydration reaction consumes a smaller quantity of water with the progression of time. This results in a gradual increase in the disparity between the growth rate of the UCS and the growth rate of the suction at later stages of the curing process. Furthermore, this phenomenon will manifest at an earlier stage in the process when the cement content is increased. This is because the increase in UCS is influenced by a number of factors, such as overall structural properties and stability, in addition to suction.

Furthermore, from Fig 14, it is also obvious that thermal conductivity increase and permeability decrease shows coupled T-H behavior. It is interesting to notice that the evolution of thermal conductivity and permeability with time is reversely similar, which is mainly attributed to the fact that hydration products precipitate in the capillary result in the thermal conductivity increase and the permeability decrease, respectively. Meanwhile, it is clear in Fig 14 that, regardless of cement content, the development of CBR and thermal conductivity over time shows similar trends. This indicates there is a strong coupling behavior between CBR and thermal conductivity (i.e. M-T coupling behaviour). These phenomena can be explained by the fact that hydration products fill the capillary pores and voids result in refinement of capillary pores and decrease in porosity.

## Conclusions and summary

With an aim to reveal the influence of the cement content on the thermal (temperature, thermal conductivity)-hydraulic (permeability, suction, VWC)-mechanical (UCS, CBR)-chemical (EC) behaviour of marine clay subgrade stabilized with cement, a series of monitoring and testing experiments were performed on the cement-clay samples with various cement contents (0%, 6%, and 12%) at different curing times (1, 3, 7, 28, and 90 days). The following conclusions summarize the experiment results of this paper:

- The cement content has an important influence on the thermal, hydraulic, mechanical, and chemical properties of the cement-clay samples. The experiment results suggest that the development of these properties are significantly dependent on the combined effect of the cement content and curing time.
- The test results show that as the cement content increases, the UCS, CBR, and thermal conductivity increase, while the permeability decreases. The reason for this is that a larger amount of cement generates more hydration products give rise to the refinement of pore structure and decrease in porosity in cement-clay mixtures. Also, the stress-strain curves present that the cement-clay samples with more cement have the characteristics of. higher stiffness and more brittle deformation as a result of more hydration products formed in the samples.
- The monitoring results show that the VWC, T, and EC increase to a peak value initially and reduce afterward with time. However, it should be emphasized that with an increase in cement content, the peak EC and peak temperature increase while the peak VWC decrease. This is because of that more cement can facilitate hydration reactions result in more ions are released and more moisture is consumed as well as producing more heat.
- The results of this investigation indicate that the THMC properties of cement-clay are strongly coupled. The hydration process plays a significant role in THMC behavior. The important internal mechanism that affects the short-term THMC behavior of cement-clay structure includes cement hydration, self-desiccation, and heat development.

Future research should investigate the water stability, corrosion resistance, and volume change of clay reinforced by cement. Meanwhile, the application of the research results to practical engineering should also be considered. Furthermore, the researcher could investigate the THMC behavior for other types of binders and thus get the optimum ratio and optimum type of binder for clay reinforcement.

## Supporting information

S1 Data Fig 1 grain size clay. (XLSX)

S2 Data Fig 5 permeability vs time vs cement content. (XLSX)

S3 Data Fig 6 suction vs time vs cement content-1. (XLSX)

S4 Data Fig 7 VWC vs time vs cement content. (XLSX)

S5 Data Fig 8 UCS vs. time vs. cement content. (XLSX)

S6 Data Fig 9 CBR vs. time vs. cement content. (XLSX)

S7 Data Fig 10 stress-strain vs time vs cement content. (XLSX)

S8 Data Fig 11 temperature vs time vs cement content. (XLSX)

S9 Data Fig 12 thermal conductivity vs. time vs. cement content. (XLSX)

S10 Data Fig 13 EC vs time vs cement content. (XLSX)
